# Tuning the properties of polyhydroxybutyrate films using acetic acid via solvent casting

**DOI:** 10.1038/srep17884

**Published:** 2015-12-07

**Authors:** Preetam Anbukarasu, Dominic Sauvageau, Anastasia Elias

**Affiliations:** 1 Department of Chemical and Materials Engineering, University of Alberta, 9211 116 St NW, Edmonton, AB, T6G 1H9, Canada

## Abstract

Biodegradable polyhydroxybutyrate (PHB) films were fabricated using acetic acid as an alternative to common solvents such as chloroform. The PHB films were prepared using a solvent casting process at temperatures ranging from 80 °C to 160 °C. The crystallinity, mechanical properties and surface morphology of the films cast at different temperatures were characterized and compared to PHB films cast using chloroform as a solvent. Results revealed that the properties of the PHB film varied considerably with solvent casting temperature. In general, samples processed with acetic acid at low temperatures had comparable mechanical properties to PHB cast using chloroform. This acetic acid based method is environmentally friendly, cost efficient and allows more flexible processing conditions and broader ranges of polymer properties than traditional methods.

Polyhydroxybutyrate (PHB) is a polymer of bacterial origin that can be broken down by enzymes known as PHB depolymerases^1^,^2^,^3^. Pure PHB can be degraded by a variety of enzymes over a broad range of temperatures, resulting in non-toxic degradation products^4^. It is a truly biodegradable and food-safe alternative to petroleum-based polymers. PHB has the potential for use in medical applications^5^,^6^ and food packaging materials^7^. Also, PHB can be functionalized and chemically modified^6^,^8^ to form self-assembled micelles^9^ and gels that have good biocompatibility and biodegradability.

Despite these advantages, the challenges in processing PHB into flexible, thin films is one of the main factors that prevent its widespread application^10^. Its high melting point (~175 °C to 180 °C) and low degradation temperature (~220 °C) limit the possibility of thermal processing to prepare PHB films. Approaches such as heat treatment, co-polymerization^11^,^12^,^13^, blending^14^,^15^ and the addition of plasticizers^16^ have been used to improve the thermal processability. By using a combination of approaches mentioned above, PHB can be extruded, rolled or pressed into films having good mechanical properties.

Thermal processing assisted by additives is the most cost effective and industrially relevant approach for large-scale production of PHB films. However, most of the additives that improve thermal processing can also reduce biodegradation rates, increase cost, generate toxic degradation products^17^,^18^ or cause health hazards related to leaching of plasticizers^13^,^14^,^15^,^16^,^17^,^18^,^19^,^20^,^21^. Recent works are exploring eco-friendly plasticizers^22^,^23^, green polymer blend, composites^24^,^25^ and additives^26^ to overcome these drawbacks, but some of the issues with using additives still exists. Films produced by thermal processing can also have limited flexibility and optical clarity, even in the presence of plasticizers and additives. Such limitations are acceptable in some applications, such as compostable bags and disposable containers. However, for other specialized applications – such as bio-medical implants and optical films, which require PHB with properties including high porosity, low thickness and optical clarity – alternative processing routes that offer more flexibility in processing conditions and PHB properties are necessary.

One such approach for fabricating PHB films is to use a solvent casting process^27^, which involves dissolving the polymer in a suitable solvent and evaporating the solvent to obtain a high-quality film. Solvent casting enables tunability of mechanical and optical properties of the film through the variation of processing parameters such as solvent casting time and temperature. The solvent casting process is capable of producing ultra-thin films that have high optical clarity and porous films that can degrade rapidly in physiological conditions. Despite these advantages, the added cost and hazards that come with solvents has limited solvent casting to niche applications such as cellulose triacetate films for photographic sheets^28^ and polyvinyl alcohol films for polarizers in liquid crystal displays^29^. A relatively safe and cost-effective solvent can bring down the cost of solvent casting and enable the production of high-quality PHB films through this route.

To produce continuous films that have good mechanical properties, a compatible solvent that has a similar solubility parameter to PHB is necessary. A previous study revealed that only a few solvents are suitable candidates for PHB, of which chloroform is one of the most compatible and most commonly used^30^,^31^. However, previous reports have rated chloroform as one of the most damaging chemical to the environment and human health^32^,^33^,^34^. In addition, the high affinity of PHB to chloroform can cause traces of chloroform to remain in the polymer even after long aging times, which could prove to be a health risk in medical implants and food packages. The potential for PHB to be used in food packaging and for medical applications makes it even more desirable to find food-safe and risk-free solvents.

The present work explores acetic acid as a solvent to dissolve and process PHB through solvent casting. Acetic acid is cost effective, safe to handle and easy to recover, as demonstrated by its extensive use in the synthesis of polymers such as cellulose acetate and polyvinyl acetate^35^. Although acetic acid is not the most compatible solvent for PHB, its high boiling point (118 °C) enables dissolution and processing of PHB at elevated temperatures. The incompatibility of acetic acid also allows us to control both the microstructure and properties of the films by taking advantage of the phase separation and crystallization behavior exhibited by the PHB/acetic acid system.

Some previous works have used a mixture of dilute acetic acid and an organic solvent to blend PHB with polymers such as chitosan^36^. Other works have used a combination of acetic acid and organic solvents (typically dimethyl sulfoxide) to conjugate polymeric side chains to PHB^37^,^38^. However, in all these instances, the dilute acetic acid was used to dissolve the combining polymer (chitosan, cellulose) and to assist blending or conjugation, while organic solvents such as chloroform or hexafluoro-2-propanol were used to dissolve the PHB prior to blending. To our knowledge, glacial acetic acid by itself has never been used to dissolve and produce PHB films.

In this work, we present the impact of the solvent casting processing conditions on the thermal behavior, crystallinity, mechanical properties and surface roughness of PHB films.

## Results and Discussion

### Purity and composition of as-received PHB

We determined the chemical composition and melting point of as-received PHB using x-ray photoelectron spectroscopy (XPS), CHNS analysis, and differential scanning calorimetry (DSC). The XPS results showed that the as-received material contained ~1 wt. % Si, which likely remained in the sample as impurity after the pelletization of PHB. We found that the C:O ratio of the as-received polymer was within 1% from the theoretical ratio (C:O ratio of 1.5) of pure PHB. These numbers are consistent with a PHB purity of 98-99%. The melting point of the as-received PHB at a heating rate of 10 °C/min was found to be ~180 °C, which agrees well with the values reported for PHB in the literature^22^,^23^.

### Appearance of PHB films

**Figure 1:**
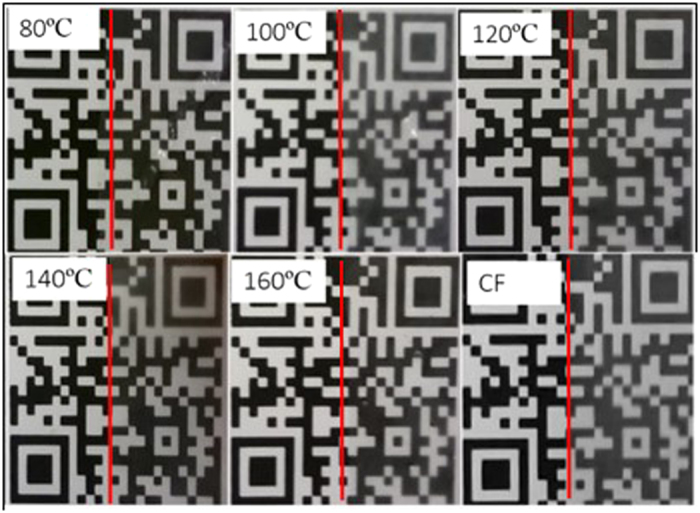
Images of PHB films processed at different temperatures overlaid on the right side (separated by red lines) of a printed pattern, demonstrating the translucency of PHB films. Results from a film processed using chloroform (CF) is shown for comparison. The film thickness of each sample was 40 ± 10 μm.

PHB films prepared at different temperatures using acetic acid as a solvent were translucent. Figure 1 show images of PHB films – prepared at various temperatures – placed on a printed pattern. The samples processed at 80 °C, 140 °C and 160 °C were more transparent than the ones processed at 100 °C and 120 °C. The samples prepared using chloroform were found to have similar optical transmittance to samples prepared at 100 °C and 120 °C.

**Figure 2:**
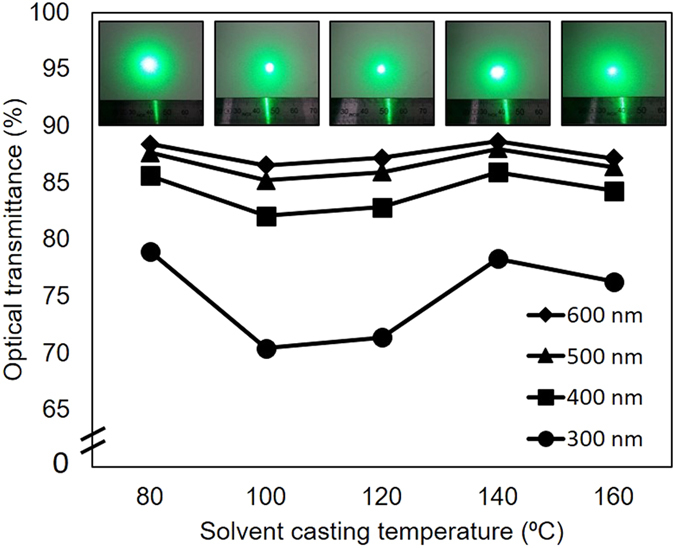
Optical transmittance vs. solvent casting temperature. The transmittance values were obtained at wavelengths of 600 nm, 500 nm, 400 nm and 300 nm. The inlay shows the images of transmitted laser beam after passing through the PHB films, arranged from lowest processing temperature (left) to highest processing temperature (right).

Figure 2 shows a plot of optical transmittance of the film at different incident light wavelengths vs. solvent casting temperature. All films had relatively high optical transmittance (>82%) in the visible portion of the spectrum. The optical transmittance at all wavelengths was found to follow a parabolic trend, with lowest (80 °C) and higher processing temperatures (140 °C and 160 °C) resulting in films with higher optical transmittance. The films processed at intermediate temperatures had the lowest transmittance, indicating that they scatter, absorb and/or reflect the most light. As these films were observed to have the most uneven surfaces, this reduced transmittance can be attributed mainly to light scattering from the surface. The inlay in Figure 2 shows images of a transmitted laser beam (λ = 532 nm) after it passed through the PHB films, cast at different temperatures. These images show that the samples prepared at 100 °C and 120 °C had a relatively small specular beam and substantial scattering. This behavior is in agreement with our observations of the uneven and cloudy appearance of these films.

The cloudy appearance can be attributed to scattering of light at the interface of crystalline-amorphous regions, and the presence of residual solvent – which can remain in samples even after long aging periods. The concentration of residual solvent is expected to be higher in samples processed from chloroform than acetic acid since chloroform interacts more strongly with PHB than acetic acid^30^.

**Figure 3:**
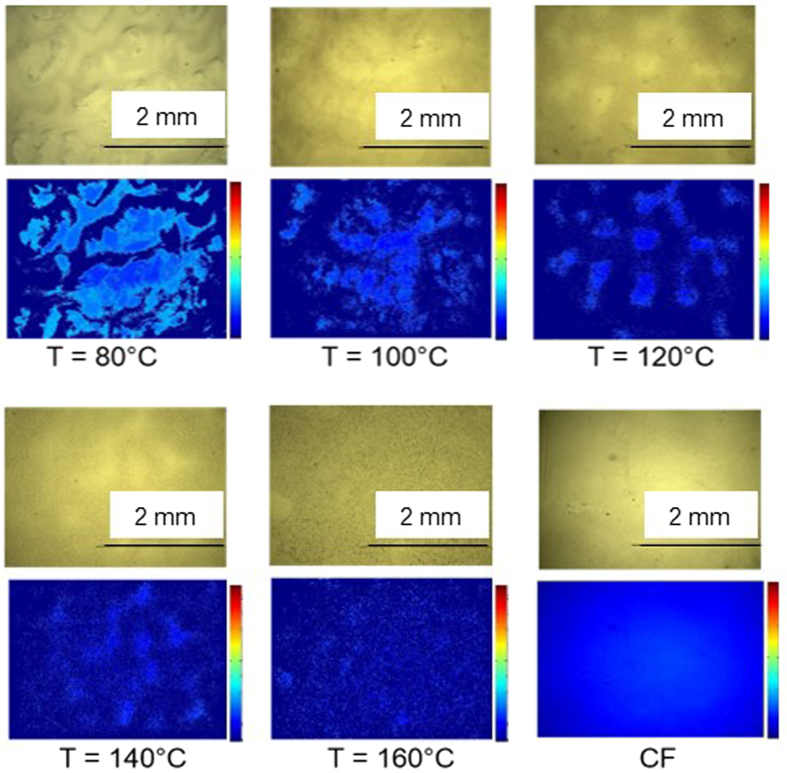
Stereomicroscope images of the PHB films solvent cast at different temperatures using acetic acid (AA) as a solvent. False-colored images (in blue) show the undulations and macroscopic features on the surface, which indicate the presence of two distinct regions in the samples. A sample processed with chloroform (CF) as the solvent is shown for comparison.

We also found that the samples prepared at high temperatures were homogenous while samples prepared at lower temperatures had a patchy appearance. Figure 3 shows stereomicroscope images of samples prepared using acetic acid. False-colored images are also shown to emphasize the undulations and features on the surface. The images indicate the presence of two distinct regions in the films, which were more apparent in the samples prepared at lower temperatures. Increasing the processing temperature resulted in more homogenous surfaces. Samples prepared at 160 °C were very smooth and had no undulation on the surface.

The variations in appearance and the presence of two regions in the samples can be explained by the fact that acetic acid, thermodynamically speaking, is a “poor” solvent for PHB^30^. This can lead to solvent/polymer phase separation and the formation of polymer-rich and polymer-poor zones in the sample as the solvent evaporates^39^. The polymer-rich regions – by virtue of having more polymer per unit volume – will have a higher average thickness than polymer-poor regions. Therefore, the polymer-rich zones can appear to be cloudier than the polymer-poor zones after complete evaporation of the solvent. Such local segregation of polymer-rich and poor regions can influence the localized solvent evaporation rate and polymer concentration, which in turn can affect properties such as the crystallization behavior, the thermal and mechanical properties^39^.

In contrast, the chloroform samples, which were cast at room temperature, had a much smoother appearance, owing to the fact that the solvent evaporation rate was much slower. The higher chemical compatibility of PHB and chloroform also limits the extent of phase separation and the consequent roughening of the surface due to mismatch stresses.

### Crystallinity

**Figure 4:**
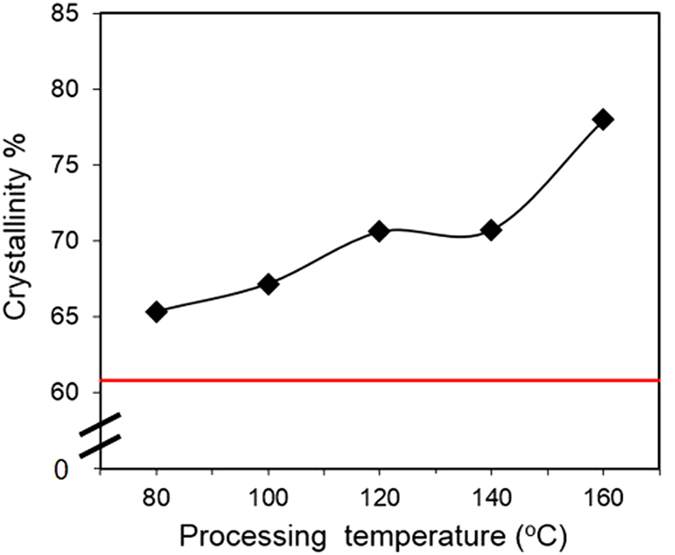
Percent crystallinity with respect to the processing temperature. The straight red line indicates the crystallinity of PHB prepared using chloroform at room temperature.

Figure 4 shows a plot of crystallinity percentage (estimated from X-ray diffraction (XRD)) as a function of processing temperature and compared to a control sample prepared at room temperature using chloroform as a solvent. The analysis of XRD diffraction peaks revealed that the crystallinity of PHB prepared using acetic acid ranged from 64% at 80 °C to 78% at 160 °C; whereas, the crystallinity of PHB processed with chloroform was approximately 60.5%. The use of glancing incidence angle and deconvolution function to calculate the contribution of amorphous regions in the polymer introduces peak broadening, and systematic instrumental error in the crystallinity values obtained using this method. Therefore, the crystallinity values are suitable only for comparison with other samples studied in this work and could deviate from absolute crystallinity values. Nonetheless, a clear trend is seen: all samples prepared using acetic acid have a higher crystallinity than the sample prepared with chloroform. This trend can be attributed to two main factors: (1) acetic acid-processed samples were cast at higher temperatures than the chloroform samples. Therefore, more thermal energy was available for the formation and growth of crystalline structures. (2) Chloroform is a “better” and more compatible solvent for PHB; residual solvent in the sample can increase polymer chain mobility and suppress crystal growth. The PHB samples also exhibited increased crystallinity with increased processing temperature, reflecting that more thermal energy resulted in a more ordered structure.

**Figure 5:**
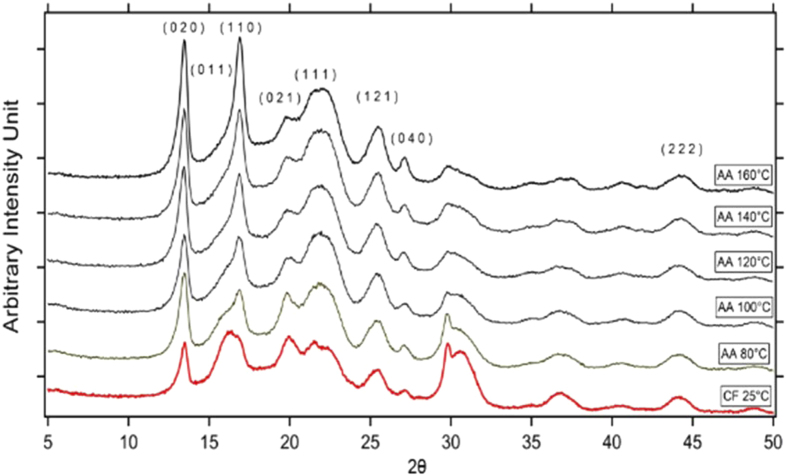
Combined XRD plot of PHB processed in acetic acid (AA) at different temperatures. The pattern obtained from PHB prepared with chloroform (CF) is shown in red for comparison.

Further examination of the structure of the films by XRD revealed that not only the percent crystallinity varied as a function of temperature, the type of crystals formed varied as well. A combined plot of XRD pattern of PHB films prepared at different solvent casting temperatures using acetic acid and chloroform as a solvent is shown in Figure 5. A general increase in intensity is seen as a function of processing temperature, reflecting the previous results.

Peaks corresponding to orthorhombic crystal planes (020), (110), (021), (111), (121), (040) and (222) at 2θ values of 13.5°, 16.85°, 19.8°, 21.4°, 25.5°, 27.2° and 44°, respectively, were found to be similar for all samples. However, the peaks corresponding to (011) at 2θ = 16.2° had much lower intensity in all acetic acid-processed samples than in chloroform-processed PHB. This indicates that the (011) plane orientation is suppressed when PHB is processed using acetic acid at higher temperatures, suggesting the presence of a preferred orientation of crystals within the samples. The lattice parameters and the peak locations for all the samples were found to be similar, indicating that the orthorhombic PHB crystals did not change substantially with increasing processing temperature. These outcomes agree well with results previously reported in the literature^40^,^41^.

### Thermal degradation

One important consideration when choosing acetic acid as a solvent for PHB is the possibility of polymer degradation through acid hydrolysis during processing. PHB is known to degrade into smaller units by random scission of ester bonds when exposed to acidic solutions^42^. Moreover, the use of high processing temperatures can accelerate this reaction and decrease the thermal stability of the sample. Therefore, we analyzed the extent of PHB degradation caused by acetic acid using thermogravimetric analysis (TGA).

**Figure 6:**
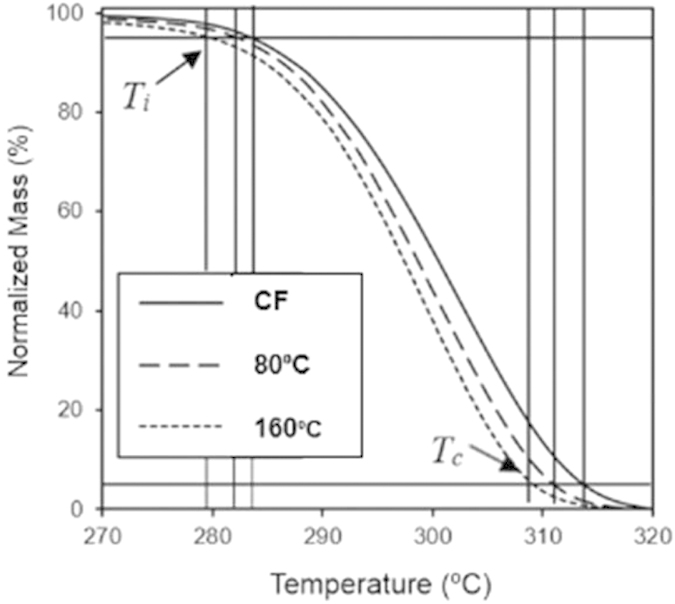
TGA plot of PHB films prepared under different conditions: solvent cast in chloroform (CF), solvent cast in acetic acid at 80 °C, and solvent cast in acetic acid at 160 °C. The upper and lower horizontal lines correspond to 95% normalized mass and 5% normalized mass respectively, while the vertical lines indicate the thermal degradation onset temperature (*T*_*i*_) and the complete degradation temperature (*T*_*c*_) of each sample.

Figure 6 shows a combined plot of TGA carried out on samples solvent cast using acetic acid at two different temperatures compared with samples cast using chloroform. The figure also indicates the temperature at which the samples lost 5% of their mass (thermal degradation onset temperature (*T*_*i*_)) and the temperature when samples lost 95% of their mass (complete degradation temperature (*T*_*c*_)). We used *T*_*i*_ and *T*_*c*_ of the samples to determine the effect of acetic acid processing on PHB. We also obtained the TGA profile of PHB prepared with chloroform for comparison with acetic acid-processed samples. The samples cast from acetic acid had lower *T*_*i*_ and *T*_*c*_ than the sample cast from chloroform. These results indicate that the use of acetic acid as a solvent and higher casting temperatures can cause mild hydrolysis and degradation of PHB. We found that the *T*_*i*_ and *T*_*c*_ were marginally lower for the 160 °C sample (279 °C and 308 °C, respectively) compared to the 80 °C sample (283 °C and 312 °C, respectively). The slope of the weight loss curves with respect to temperature was found to be very similar (the difference in slope is close to the instrumental limit) for samples prepared at 80 °C and 160 °C. The samples prepared using acetic acid were dissolved at elevated temperature for ~1 hour, while the solvent casting process itself lasted only a few minutes, which explains why both the slopes and the temperature at which the degradation is complete (100% mass loss) are similar for these samples. In contrast, the sample cast from chloroform had a lower slope and reached 100% mass loss at a slightly higher temperature. These factors also indicate that the samples prepared using acetic acid as solvent underwent slight degradation (and likely experienced a small decrease in molecular weight mainly during the dissolution process), while the solvent casting step did not cause much change because of the short thermal exposure time. Overall, the acetic acid processing route used in this work did not cause substantial degradation and more importantly allowed for the production of free-standing thin films that have good thermal stability.

### Melting behavior

**Figure 7:**
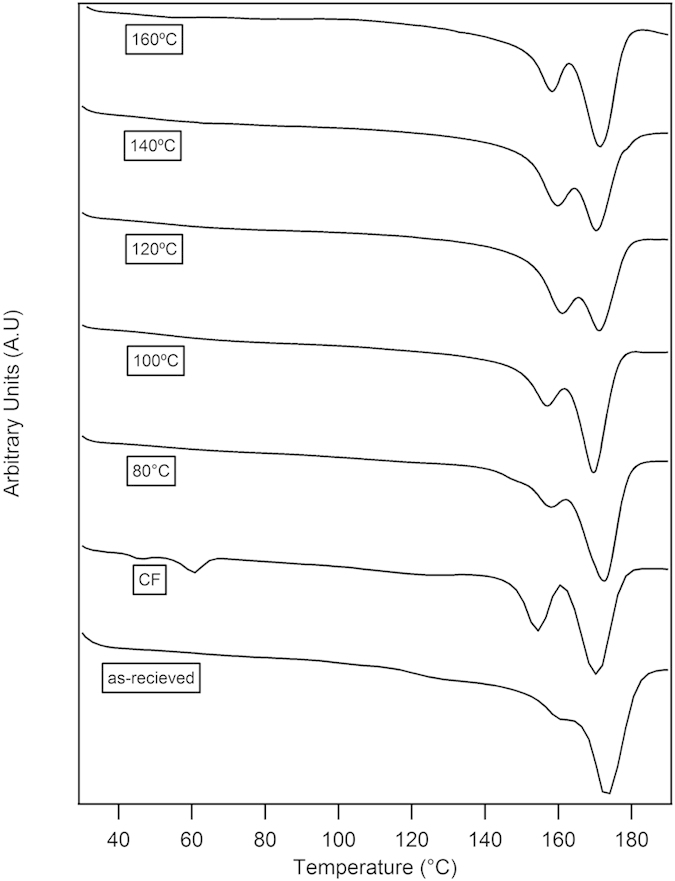
Combined plot of the DSC melting curves for PHB samples prepared at different temperatures in acetic acid (AA). The as-received sample and sample solvent using chloroform (CF) are also shown for comparison. All samples were run at a scan rate of 20 °C /min (endothermic down).

The melting behavior of the PHB films was determined using DSC. The melting curves of the films prepared at different temperatures are shown in Figure 7. The melting endotherm consisted of two distinct peaks for all PHB samples while the as-received material exhibited a large peak with a small shoulder at a lower temperature. The double melting behavior is quite common in polymeric materials and has been explained based on two theories: namely, the melting and recrystallization model^43^ and the double lamellar thickness population model^44^,^45^. Previous works have reported the double-melting behavior for PHB and have shown that it occurs according to the melting and recrystallization model as observed by the change in the shape of the endotherms at different heating rates^46^,^47^.

The magnitude of the low-temperature endotherm is directly related to the amount of as-formed metastable crystals while the high-temperature endotherm corresponds to the melting of ordered crystals. From the DSC curves, we observed that the magnitude of the first endotherm followed a parabolic trend (the magnitude of the endotherm increased from 80 °C to 140 °C but decreased for sample prepared at 160 °C) with increasing processing temperature. The presence of such metastable as-formed crystals can be attributed to the phase-separation behavior exhibited by the PHB/acetic acid system. The presence of polymer-rich and polymer-poor regions cause localized variations in polymer chain mobility (polymer-rich regions have low mobility) and solvent evaporation rate (polymer-rich regions have slower evaporation rate), which in turn can influence the nature of ordered crystals formed during the solvent evaporation process. The mechanism of phase separation can also influence the orderliness of the crystals, with nucleation and growth mechanism allowing for the formation of highly ordered crystals and spinodal decomposition resulting in relatively disordered metastable crystals. The samples prepared at lower temperatures (80 °C and 100 °C) were more likely to phase separate by nucleation and growth, since the rate of change of polymer solution concentration is much lower and more time was available for the growth of the crystals. Therefore, a smaller fraction of metastable crystals was present, as shown by the relatively small amplitude of the first melting endotherm. As the temperature increased (e.g. to 120 °C and 140 °C), rapid de-mixing due to spinodal decomposition is expected to be the dominant mechanism of crystal growth, which results in the formation of more metastable crystals (and a relative increase in the magnitude of the first peak with respect to the second). As the temperature increased further (160 °C), the phase separation effect became less important because of the high solvent evaporation rate, and the availability of large amounts of thermal energy enabled the formation of highly ordered crystals at the expense of metastable crystallites (resulting in a larger second peak and a smaller first peak).

We also observe that the DSC heating curve for the sample prepared using chloroform consists of two melting peaks at approximately the same locations as the other samples. However, an additional endotherm at ~ 60 °C was apparent. This peak matches with the evaporation temperature of chloroform, indicating that a small quantity of chloroform remained in the sample even after a long aging time. This could limit the applicability of chloroform-cast PHB films for use in packaging and other applications that involve direct contact with food or the body. The strong interaction between PHB and chloroform can also affect crystal formation. Therefore, the underlying crystallization conditions and structure of the sample solvent cast using chloroform would be much different from the samples cast using acetic acid. Nonetheless, as for the samples cast from acetic acid, a large metastable peak is seen for these samples as well.

These results suggest that all the samples (including those cast from chloroform) contain a mixture of stable and metastable crystallites, with intermediate processing temperatures resulting in more metastable crystals. Despite the fact that the samples cast from chloroform contained a significant fraction of both types of crystals, these samples were much smoother and uniform in appearance than the samples cast from acetic acid at low temperatures. The large fraction of metastable crystals in chloroform-processed samples can be explained by the low processing temperature, which limits the amount of thermal energy available for the formation of ordered crystals. The uniform surface, on the other hand, can be attributed to slow solvent evaporation rate and higher compatibility of PHB and chloroform. We expect that the proportion, quantity, and relative size of the different crystal types can influence the mechanical properties of the samples since stable, and metastable crystals are expected to have different mechanical properties. These properties are characterized in the next section.

### Mechanical properties

**Figure 8:**
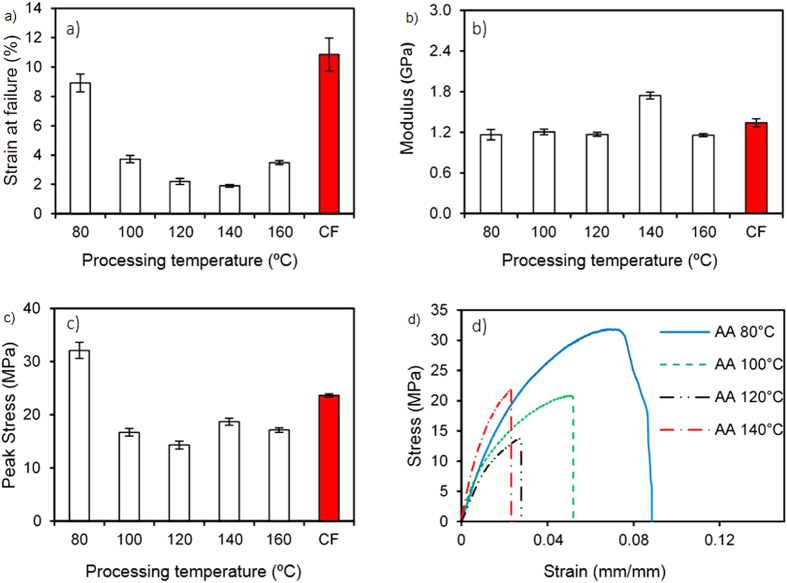
Mechanical characterization of PHB films prepared in acetic acid (AA) at different casting temperatures. (**a**) Strain to failure vs. casting temperature, (**b**) Elastic modulus vs. processing temperature, (**c**) Ultimate tensile stress vs. processing temperature, (**d**) Representative stress-strain curves. Average values and standard error are shown in (**a–c**) and are based on at least 4 measurements. Results from samples cast with chloroform (CF) are also shown for comparison.

Figure 8 shows plots of mechanical properties of PHB films solvent cast in acetic acid at different temperatures. Results from samples cast with chloroform are also shown for comparison. In general, both the strain to failure and peak tensile stress were much higher for the films cast at 80 °C than at the higher processing temperatures. In contrast, the elastic modulus did not change substantially as a function of temperature, except at a processing temperature of 140 °C. The decrease in strain to failure correlates well with the increase in crystallinity with processing temperature. The presence of amorphous regions (above the glass transition temperature) lends flexibility to the polymer. With increasing processing temperature, more crystallites are formed at the expense of amorphous regions, which results in increased brittleness of the polymer^48^. The presence of a large number of crystallites has been shown to vitrify amorphous polymer chains. This vitrification further embrittles PHB and lowers the strain to failure and ultimate tensile stress^49^,^50^,^51^. We attribute an almost constant elastic modulus to the low strain (0.5 mm/min) rate used for the tensile test. Low strain rates can have an effect analogous to deforming a polymer at elevated temperatures, where the elastic modulus decreases and converges to a minimum for a given strain rate^52^. Low strain rates can also accommodate changes in the orientation of crystallites, which enables amorphous chains to elongate reversibly, especially at small strains (~1%).

Interestingly, the samples prepared at 160 °C deviated from the general trend, exhibiting a higher strain at failure than samples cast at 120 °C and 140 °C. This behavior could be attributed to partial melting and the consequent stress relaxation of PHB at 160 °C. Although this processing temperature was lower than the melting point of PHB (~180 °C), the melting process can begin at temperatures as low as 150 °C, enabling a small proportion of the sample to melt and accommodate the stresses generated at the crystalline-amorphous interfaces. The stress relaxation effect can explain the increase in strain to failure for the samples prepared at 160 °C, despite it exhibiting much higher overall crystallinity than the rest of the samples. The relatively high strain to failure and ultimate tensile stress of samples processed at 80 °C can be explained by the low crystallinity and the possible presence of trace quantities of acetic acid (although we did not observe any evidence of residual solvent from the TGA heating curves). In the latter case, acetic acid could have a plasticizing effect on PHB, causing an increase in strain to failure. Overall, the properties of the samples processed at all temperatures were comparable with those of the chloroform-cast films.

### Surface morphology and roughness

**Figure 9:**
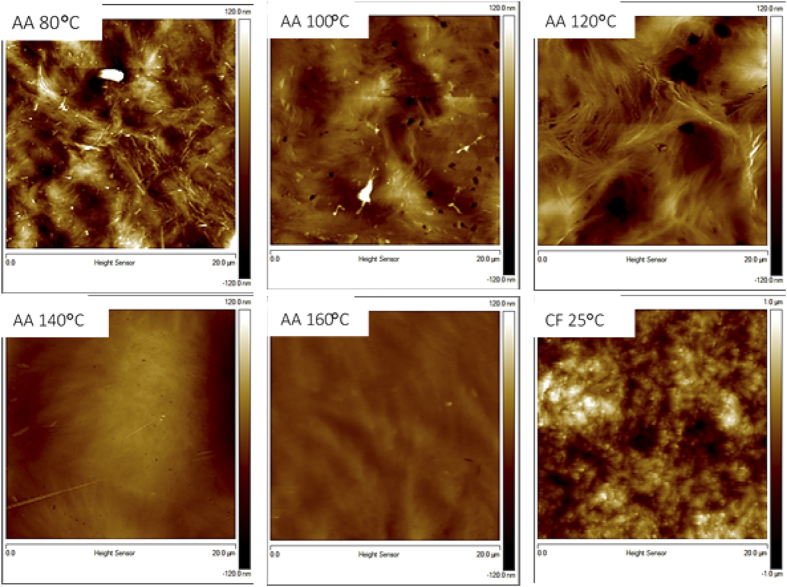
AFM scans of PHB samples processed at different temperatures in acetic acid (AA) or chloroform (CF). Scan area: 20 μm^2^. The topography scale is ±120 nm for all acetic acid-processed samples, and ±1000 nm for the chloroform-processed sample.

**Figure 10:**
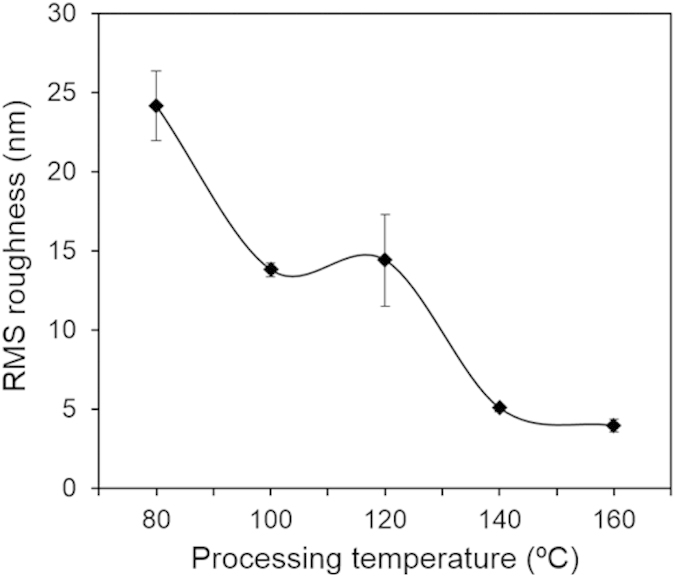
RMS roughness of PHB films solvent cast with acetic acid with respect to the processing temperature.

The surface morphology of the PHB films prepared at different temperatures is shown in Figure 9; characterization was performed on the surfaces that were cast against the glass slide. The atomic force microscopy (AFM) images show a trend of decreasing roughness with increasing processing temperature. In addition to this, the samples processed with acetic acid have contrasting surface morphology from those processed with chloroform. We found that the PHB films prepared with chloroform had rough and globular structures on their surface. The samples processed with acetic acid at low temperatures had a fibrous, needle-like structures and roughness in the range of 20 nm, whereas the samples processed at high temperatures had smooth, uniform surfaces. The needle-like structures were likely caused by small crystallites and impurities on the surface. The RMS surface roughness with respect to solvent casting temperature is shown in Figure 10 and was found to decrease progressively with increasing processing temperature. The RMS roughness of PHB prepared with chloroform was found to be in the range of 1 μm, which is over an order of magnitude greater than the samples prepared using acetic acid.

The increase in roughness with decreasing temperature can be explained by the fact that the polymer chains have less energy available to form ordered structures throughout the surface when processed at low temperatures. Therefore, more amorphous regions are present on the surface, along with the highly ordered crystallites. A large number of crystalline-amorphous interfaces can explain the increased roughness seen in PHB surface processed at room temperature using chloroform. In this case, small crystals and amorphous zones populated the surface.

The high surface roughness of chloroform-processed samples could also contribute to the cloudy appearance seen in Figure 1 since a surface with roughness comparable to the wavelength spectrum of visible light would result in more scattering. The acetic acid-processed samples, on the other hand, are expected to have much lower scattering since the surface features are much smaller than the wavelength spectrum of visible light.

## Discussion

In summary, we have shown that PHB can be solvent cast into films using acetic acid as a solvent, with only a marginal decrease in thermal stability. The different solvent casting temperatures employed in this work enabled us to control the solvent evaporation rate and cooling rate. These factors affect microscopic features namely: 1)crystallinity, 2) nature and orderliness of the crystals, and 3) fraction of stable/metastable crystals. These microscopic features lead to differences in properties.

In general, at low casting temperatures, the solvent evaporates slowly, and there is limited thermal energy available for crystallization. Films, therefore, have low crystallinity, have good mechanical properties (in terms of tensile strength and strain to failure), and reasonable optical transmittance. However, films processed at lower temperatures have rougher surfaces both at the macroscopic and microscopic scale – due to variations in thickness resulting from phase separation, and to inhomogeneities caused by the limited thermal energy available for crystallization. On the other hand, higher solvent casting temperatures yield films that are more crystalline, more transparent, and have higher surface uniformity. However, these films have relatively low tensile strength and strain. These results show that the proper selection of casting temperature and solvent evaporation rate can be used to achieve films with the desired set of properties.

**Figure 11:**
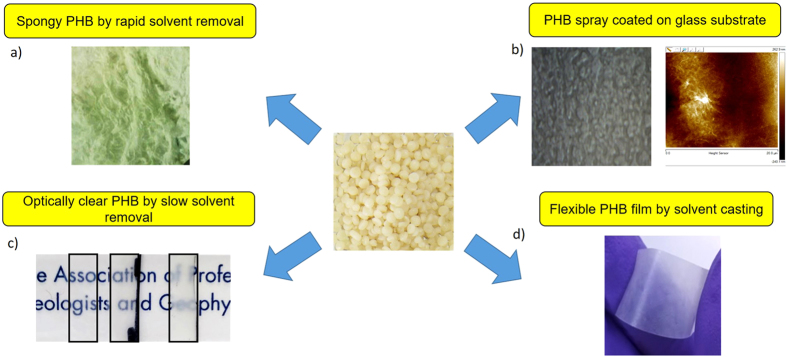
Ensemble of different forms of PHB produced using acetic acid as a solvent. (**a**) Porous PHB by rapid removal of solvent, (**b**) spray coated PHB layer on a glass substrate, (**c**) PHB thin films that have different optical transmittance. Films prepared at different acetic acid concentrations at 80 °C. Optical transmittance decreases from left to right, (**d**) Flexible PHB films prepared by solvent casting from acetic acid.

These results establish that acetic acid is a viable solvent to process PHB films. This method can easily be adapted and altered to produce PHB that has a much wider set of properties than what is possible with other processing routes. Figure 11 shows an ensemble of different forms in which PHB can be processed using acetic acid. In addition to films and sheets that have varying optical transmittance (Fig. 11c,d), we have been able to produce PHB films that have high porosity (Fig. 11a) by rapid phase separation of PHB and acetic acid by introduction of non-solvent (water in this case). Porous PHB could be useful in bio-implant and biomedical applications, because of its rapid and controllable degradation behavior. We have also been able to spray coat a thin layer of PHB (with acetic acid as solvent) on glass, paper and on other plastic surfaces using a simple airbrush. Such varied forms of PHB can find potential applications as biological scaffolds, packaging materials, sensing devices and as enzyme activity screening assays.

While all these forms of PHB can be prepared using chloroform as a solvent or using thermal processing, acetic acid as solvent provides the greatest versatility in processing conditions and PHB properties.

## Conclusion

We have demonstrated the possibility of using acetic acid as a cost-effective solvent to prepare flexible PHB films. These films exhibited optical, mechanical and surface properties similar to or better than those of films produced using chloroform, the typical solvent for PHB. The films prepared with acetic acid in 6 minutes had comparable properties to films prepared using chloroform in 24 hours. The relatively high dissolution and solvent casting temperature while requiring more thermal energy, afford the advantage of short processing times and the ability to tune the properties of the film. The crystallinity of the films can be varied through the selection of a suitable casting temperature, allowing the mechanical and optical properties of the films to be altered based on the requirement. The proposed method offers a simple route to make high quality, flexible PHB films for applications ranging from packaging to biodegradable implants.

## Materials and Method

### Polymer and Chemicals

The PHB (98%) used in the work was obtained as thermally processed pellets (BRS Bulk Bio-pellets, Bulk Reef Supply, Golden Valley, USA). The PHB pellets were washed with isopropyl alcohol to prevent microbial contamination and otherwise used as received. Acetic acid (99%) and chloroform (99%) were obtained from Sigma-Aldrich, Canada and used as received.

### PHB film preparation

**Figure 12:**
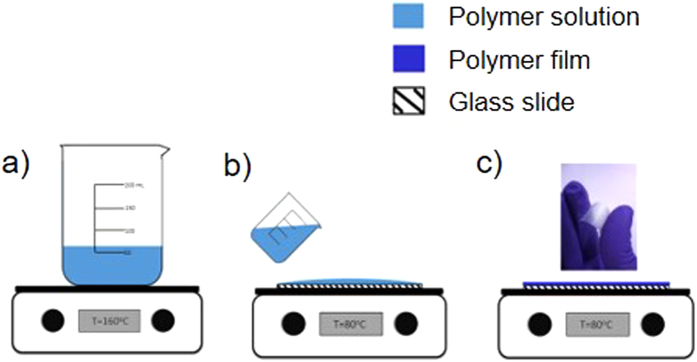
Schematic of PHB film preparation by solvent casting. (**a**) The PHB is dissolved in acetic acid at 160 °C, (**b**) the solution is poured onto a glass slide held at the casting temperature, (**c**) samples are baked for 3 or 6 minutes (depending on the casting temperature) at which point films can be removed from the slide.

The PHB pellets were mixed with acetic acid and heated to boiling in a covered beaker under constant stirring until the sample was completely dissolved (typically ~40 to 60 minutes) (Fig. 12a). A polymer solution with a concentration of 0.05 g/ml of PHB in acetic acid was used to prepare all test films. Approximately 4.5–5 ml of polymer solution – previously brought to the required casting temperature – was poured on a pre-heated glass slide (70 mm X 35 mm) maintained at the required casting temperature (80 °C, 100 °C, 120 °C, 140 °C or 160 °C) (Fig. 12b). Films were obtained after complete evaporation of the solvent (Fig. 12c). The solvent casting time was varied based on the boiling point of acetic acid (118 °C): samples cast at temperatures above 118 °C were dried for 3 minutes, whereas the samples cast at temperatures below 118 °C were dried for 6 minutes to ensure complete removal of solvent. The prepared film samples were stored at room temperature for 24 hours prior to characterization.

As a comparative sample, PHB was solvent cast using chloroform. PHB was dissolved in chloroform at 70 °C for 1 hour and then poured onto glass slides at room temperature; the solution was dried at 25 °C for 24 hours to prepare film samples. The samples were aged for five days at atmospheric pressure and room temperature, and then vacuum dried for 3 hours to remove most of the residual chloroform.

### Elemental analysis

The chemical composition of the as-received PHB material was characterized using an Axis Ultra (Kratos Analytical) X-ray photoelectron spectrometer (XPS) and a Carlo Erba EA1108 Elemental Analyzer for CHNS and oxygen detection. XPS was carried out over binding energy values ranging from 0 eV to 1500 eV, at a scan rate and energy step of 2 eV/second and 400 meV, respectively. The areas of peaks corresponding to given elemental bonds were used to determine the chemical composition of the sample. CHNS analysis was carried out following a modified form of the Pregl-Dumas technique^53^. The C:O and C:H ratios were used to confirm the purity of the as-received material.

### Optical transmittance

The optical transmittance of the PHB samples was characterized using a Perkin-Elmer Lambda 900 NIR-UV-Vis spectrometer with an integrating sphere and optical bench attachment. The PHB samples were mounted in front of the integrating sphere perpendicular to the path of the incident light beam so that all the light transmitted through the sample was captured by the detector. The transmittance was determined for wavelengths ranging from 300 nm to 800 nm.

### X-ray diffraction (XRD)

The crystallinity was determined using a Rigaku X-ray diffraction (XRD) system in glancing incidence angle mode. The scan was carried out between 5° and 60° at a rate of 2°/min using Cu Kα X-rays at 44 kV. An Ultima VI goniometer fitted with a thin film attachment was used to characterize the samples. An X-ray beam spot of 5 mm diameter was used for all scans. The samples were attached to a glass slide and kept as flat as possible. Baseline correction and de-convolution of the amorphous halo from the actual XRD pattern were carried out using Igor Pro 6.35A5. The area under the crystalline peak was used as a measure of overall crystallinity of samples cast at different temperatures. The crystallinity percentage with respect to solvent casting temperature was plotted and compared with results obtained using chloroform.

### Thermogravimetric analysis (TGA)

The extent of thermal degradation due to dissolution and processing of PHB in acetic acid was determined using a Mettler Toledo TGA/DSC 1 system. TGA scans were carried out from 25 °C to 380 °C, at a heating rate of 10 °C/minute. The temperature of complete degradation and the range of temperatures over which the degradation occurred were used to determine the extent of degradation.

### Differential scanning calorimetry (DSC)

The melting temperature and the shape of the melting endotherm of the base material and processed samples were determined using a differential scanning calorimeter (DSC). The DSC was calibrated using indium and zinc standards. At least 5 mg of sample were used for each run, and closed aluminum pans were used for the samples. DSC analysis was carried out from 25 °C to 195 °C at a heating rate of 10 °C/min for the as-received material. While, the heating rate was maintained at 20 °C/min for the DSC runs of all solvent cast films. This was done to limit the extent of recrystallization and thermal effects during the heating cycle.

### Tensile testing

The PHB films were sectioned into rectangular samples of dimensions 25 mm ×5 ± 0.5 mm ×55 ± 8 μm (length x width x thickness). An Instron 5943 tensile tester with a 1 kN load cell was used to carry out all tests. Data was collected at a strain rate of 0.5 mm/min. For each casting temperature, at least four samples were tested, and the average (with standard error) was plotted with respect to the processing temperature. The elastic modulus was obtained by measuring the slope over the linear region of the stress-strain curve.

### Atomic force microscopy (AFM)

The surface morphology and the root mean square roughness (RMS) of solvent cast PHB surfaces were determined using a Bruker Nano Dimension Edge atomic force microscope (AFM). The AFM was operated in tapping mode using a tip with a spring constant of 40 N/m. Characterization of each sample was carried out on the smooth, cast surface obtained when the sample was peeled away from the glass slide. The RMS roughness over an area of 3 μm^2^ was obtained at five relatively flat regions free from any visible voids.

## Additional Information

**How to cite this article**: Anbukarasu, P. *et al.* Tuning the properties of polyhydroxybutyrate films using acetic acid via solvent casting. *Sci. Rep.* **5**, 17884; doi: 10.1038/srep17884 (2015).
